# Circulation of foot-and-mouth disease serotypes, risk factors, and their effect on hematological and biochemical profiles among cattle and buffalo in Quetta, Balochistan, Pakistan

**DOI:** 10.14202/vetworld.2024.329-336

**Published:** 2024-02-07

**Authors:** Daud Khan, Irfan Shahzad Sheikh, Asad Ullah, Khushal Khan Kasi, Mohammad Zahid Mustafa, Zia Ud Din, Ismail Anwar, Niamatullah Kakar, Abdul Waheed

**Affiliations:** 1 Department of Livestock and Dairy Development, Balochistan, Pakistan; 2 Center for Advanced Studies in Vaccinology and Biotechnology, University of Balochistan, Quetta, Pakistan; 3 Department of Natural and Basic Sciences, University of Turbat, Pakistan; 4 Department of Livestock and Poultry Production, Bahauddin Zakariya University Multan, Pakistan

**Keywords:** buffalo, cattle, enzyme-linked immunosorbent assay, foot-and-mouth disease, Quetta, serotype

## Abstract

**Background and Aim:**

Foot-and-mouth disease (FMD) is an infectious disease of cloven-hoofed animals, including buffalo, cattle, sheep, goats, and pigs, causing major economic losses to the local farmers and, overall, to the national economy of the country. This study aimed to detect FMDV serotypes in year-round FMD outbreaks, hematological and biochemical changes, and oxidative stress in FMDV-infected cattle and buffaloes in the district of Quetta, Balochistan, Pakistan, and the socioeconomic impact of FMD outbreaks on farmers.

**Materials and Methods:**

We conducted a cross-sectional study in the district of Quetta, Balochistan, Pakistan, where FMD virus (FMDV) serotypes were detected by enzyme-linked immunosorbent assay (ELISA). Hematological, biochemical, and oxidative analyses were performed by analyzing the blood of FMDV-infected and non-infected animals. Information on the associated risk factors was obtained through a structured questionnaire by interviewing farmers in each FMD-affected farm.

**Results:**

Thirty-four out of 38 farms (89%, 95% confidence interval [CI]: 75%–97%) were positive for FMD by ELISA. Higher FMD infection was detected in farms with a herd size of <50 animals (50%, 17/34), followed by >100 animals (32%, 11/34) and 51–100 animals (18%, 6/34). Fifty-seven percent (114/200, 95% CI: 50%-64%) of animals were positive for FMD. Of these, 61% (69/114) were cattle and 39% (45/114) were buffalo. FMD positivity was higher in females (86%, 98/114) than in males (14%, 16/114) and higher in animals older than 2 years of age (52%, 59/114). On average, farmers lose U.S. dollars 3000 annually due to FMD outbreaks. Animals infected with FMDV had significantly (p ≤ 0.05) white blood cell counts and significantly (p ≤ 0.05) lower hemoglobin and total protein concentrations in buffalo and cattle, whereas infected cattle showed significantly (p ≤ 0.05) lower albumin levels. Globulin levels were lower in buffaloes infected. Alanine aminotransferase levels were lower in infected cattle (p ≤ 0.05). Creatinine levels were higher in infected buffalo (p ≤ 0.05). Urea and phosphorus concentrations were higher in FMDV-infected cattle and buffalo (p ≤ 0.05). Calcium levels were lower in infected cattle and buffalo (p ≤ 0.05). Catalase enzyme activity in infected cattle and buffaloes was significantly lower (p < 0.05). Lipid peroxidation was significantly higher in FMDV-infected cattle and buffalo (p ≤ 0.05).

**Conclusion:**

This study confirmed serotype O circulation among cattle and buffalo in year-long FMD outbreaks in the Quetta District of Balochistan. Blood analysis identified a parameter deviated from the normal level due to FMDV infection. In addition, the outbreak of FMD has a significant negative economic impact on livestock farmers.

## Introduction

Foot-and-mouth disease (FMD) is an infectious disease in cloven-hoofed animals, including buffalo, cattle, sheep, goats, and pigs. The mortality rate is low, but it has a high morbidity rate, resulting in major economic losses to local farmers and, overall, to the national economy of the country [1]. The causative agent is a single-stranded, negative-sense ribonucleic acid virus belonging to the family *Picornaviridae* and *Aphthovirus* genus. This virus has seven serotypes, namely, O, A, Asia 1, C, SAT I, SAT II, and SAT III and more than 60 subtypes [1, 2]. FMD virus (FMDV) is transmitted by inhalation of infected droplets, ingestion of contaminated vaccines, and insemination of contaminated semen. Replication of viruses mainly occurs in the lungs, oral cavity, heart, udder, and feet. Clinical signs include fever, anorexia, and blisters on the mucous membranes of the mouth, udder, and feet [2]. In addition, direct losses include low meat and milk production, loss of draught power, treatment costs, and restrictions on the export of animals and their byproducts [1]. These serotype outbreaks are not restricted to a specific geographical region [1]. It is widespread in Asia, the Middle East, and Africa. Few countries, including New Zealand, Japan, and Australia, have succeeded in eradicating this virus [2]. However, in disease-free countries, there remains a significant risk of re-establishment of this disease [3]. FMDV is endemic in Pakistan with the prevalence of Asia I, Asia A, and Asia O serotypes. From 2002 to 2005, up to 1286 FMD outbreaks have been reported in both large and small ruminants in Pakistan [4]. Outbreaks are mainly reported throughout the year. FMD has been reported in almost all parts of Balochistan [5]. In Pakistan, the estimated cost due to FMDV outbreaks is up to 21 million U.S. dollars (USD) annually [6].

Pakistan is an agricultural country. In Balochistan province, up to 47% of the provincial economy is dependent on animal husbandry practices [7]; a large number of the population is dependent on livestock for their livelihood. In Balochistan, there are 2.2 million cattle and 0.3 million buffaloes [8]. Balochistan is geographically located in the south-west part of Pakistan, bordered with Afghanistan and Iran. The continuous movement of animals across borders increases the risk of transboundary diseases. Moreover, Quetta district has a higher human population than other districts in Balochistan, and a large number of livestock are transported to this district to fulfill the protein and milk requirements. Therefore, we selected FMDV serotypes in and around Quetta district, which will be helpful in the prevention and control of FMDV outbreaks in these areas. In addition, the determination of hematological and biochemical profiles in relation to FMDV infection will be useful for determining the physiological changes in animals caused by FMDV infection.

Therefore, this study aimed to detect FMDV serotypes in year-round FMD outbreaks, hematological and biochemical changes, and oxidative stress in FMDV-infected cattle and buffaloes in the district of Quetta, Balochistan, Pakistan, and the socioeconomic impact of FMD outbreaks on farmers.

## Materials and Methods

### Ethical approval

This study was approved by the Ethics Committee of the Center for Advanced Studies in Vaccinology and Biotechnology (CASVAB), University of Balochistan Quetta, Pakistan (No. UoB/Acad/CASVAB: 058).

### Study period and location

The study was conducted from January to December 2021. This study was conducted in the Quetta district and its surroundings. Quetta district is the provincial capital of Balochistan. It is geographically located in the north-west part of the province. The climate is arid and semi-arid. According to the 2006 census, there are 11,244 cattle, 25,547 buffaloes, 163,799 sheep, and 120,384 goats in Quetta district [8].

### Study design

A cross-sectional study was conducted to detect FMDV serotypes, hematological and biochemical changes, and oxidative stress in FMDV-infected cattle and buffaloes in the district of Quetta, Balochistan, Pakistan, as well as the socioeconomic impact of FMD outbreaks on farmers in Pakistan. A farm was defined as a confined area having ≥05 cattle and/or buffaloes per herd with an intensive management system for milk or meat purposes or both. FMD-affected farms in the study area were identified based on the presence of FMD clinical signs in at least one animal on the farm. All laboratory investigations were conducted at the Center for Advance Vaccinology and Biotechnology, University of Balochistan Quetta.

### Enzyme-linked immunosorbent assay (ELISA)

Animals with clinical signs of fever and blister-like sores on lips, tongue, mouth, and between hooves and teats were selected from a livestock farm [9]. Lesions on buccal mucosa, tongue tissue, udder, or hoof wounds were collected in sterile Falcon™ tubes (Thermo Fisher Scientific, Massachusetts, USA) containing a transport medium composed of glycerol, phosphate buffer, and antibiotics and transported to the laboratory for analysis. ELISA was performed according to the manufacturer’s instructions (IZSLER, Brescia, Italy). Sandwich ELISA was used for FMDV antigen detection and serotyping of FMDV serotypes O, A, Asia 1, SAT 1, and SAT 2.

### Hematological analysis

Five milliliters (5 mL) of blood was collected from the animal’s jugular vein using a BD Vacutainer^®^ (Becton Dickinson, New Jersey, USA) in ethylenediaminetetraacetic acid anticoagulant [2, 10, 11]. Each sampling tube was labeled with a specific animal and farm identification and transferred to the laboratory for hematological analysis. A complete blood count analyzer (Medonic M series) was used to analyze hematological indices. Erythrocyte count, mean corpuscular volume (MCV), hemoglobin (Hb), hematocrit values (HCT), leukocyte count (WBCs), and mean corpuscular Hb concentration (MCHC) were the parameters studied.

### Biochemical and oxidative stress analysis

Ten milliliters (10 mL) of blood was collected from the animal’s jugular vein using BD Vacutainer^®^ without anticoagulant. Each sampling tube was labeled with a specific animal and farm identification and transferred to the laboratory for further analysis. Serum biochemistry was determined using a Microlab 300 Semi-automatic Chemistry Analyzer (Elitech, Netherlands) for alanine aminotransferase (ALT), creatinine, glucose, urea, albumin, cholesterol, globulin, phosphorus, calcium (Innoline Merck Pvt. Ltd, France), aspartate aminotransferase, MTD Diagnostics Sri. Maddaloni (CE) Italy, and total protein (Diagnostic System GmbH, Holzheim Germany). Blood samples were also analyzed to determine the oxidative status of animals. The catalase enzyme activity was determined according to the methodology described by Hadwan and Abed [12]. Lipid peroxidation was determined using the thiobarbituric acid reactive substances assay described by Feldman [13].

### Determination of risk factors

Information on the associated risk factors was obtained from a structured questionnaire by interviewing farmers in each FMD-affected farm. The first part of the questionnaire focused on farm-related variables, such as type of farm, herd size, animal quarantine practices, visits of other farm workers, keeping of animals of different age groups at the farm, farm hygiene, presence of other animal species other than bovines, and feeding method. The second part of the questionnaire focused on animal-related variables, such as species, age, sex, FMD vaccination, and body condition.

### Statistical analysis

All data were managed and recorded in Microsoft Excel 2010 (Microsoft, Washington USA). For statistical analyses, we used R (http://www.R-project.org)and R-Studio(an integrated development environment for R software for statistical computing and graphics) [14]. Binomial confidence intervals (CIs) for proportions were estimated by the exact 95% Clopper and Pearson interval method using the binom test function from the binom package in Dorai-Raj [15]. Fisher’s exact test was used for univariable analysis using the CrossTable function from the gmodels package in Warnes *et al*. [16]. The explanatory or independent variables were obtained from a moderated interview by filling out the structured questionnaire, and the FMD ELISA result was the outcome or dependent variable. The questionnaire also included questions on the annual economic losses of farmers due to FMD outbreaks. Farm management- and animal-level-related variables were separately analyzed. The farm was considered positive if at least one animal at the farm was positive for FMD by ELISA. Hematology, serum biochemistry, and oxidative status of FMDV-infected and non-infected animals were compared by independent t-test using Statistical Package for the Social Sciences (SPSS) (version 29.0.x, IBM SPSS Statistics, Chicago, Illinois, USA). Variables with p ≤ 0.05 were considered statistically significant.

## Results

### Farm-level prevalence of FMD and associated risk factors

**Figure-1:**
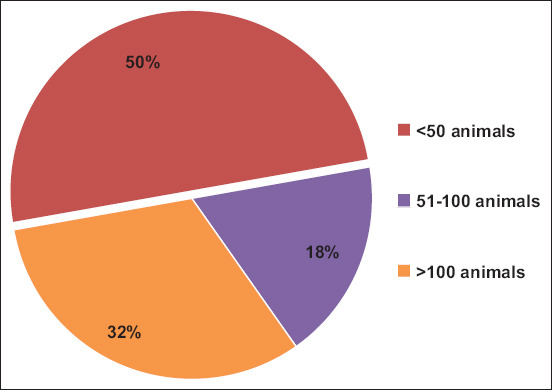
Farms positive for foot-and-mouth disease with respect to herd size.

Thirty-four out of 38 farms (89%, 95% CI: 75%–97%) were positive for FMD by ELISA. Thirty-three out of 34 FMD-positive farms (97%) were for dairy production purposes (p = 1). Thirty-seven out of 38 farms had only bovine species. All farms used stall feeding (100%). Thirty-three out of 34 FMD-positive farms (97%) had a history of worker visits to other farms (p = 1). Higher FMD infection was detected in farms with a herd size of <50 animals (50%, 17 out of 34), followed by >100 animals (32%, 11 out of 34) and 51–100 animals (18%, 6 out of 34) (Figure-1) (p = 0.255). Thirty-one out of 34 FMD-positive farms (91%) did not practice quarantining of newly purchased animals (p = 1). Among FMD-positive farms, two (6%, 2 out of 34) had poor farm hygiene (p = 1). Farmers face losses of approximately USD 3200/year due to FMD outbreaks in their farms. All farm management-related variables included in the univariate analysis showed an insignificant association with FMDV infection (Table-1).

**Table-1:** Univariable analysis of farm management-related variables (n = 38).

Variable	OR	95% CI	df	p-value
Type of farm				
Dairy (n = 37)	Ref			
Fattening (n = 1)	-	-	1	1
Herd size	-	-	2	0.255
< 50 (n = 21)				
51–100 (n = 6)				
>100 (n = 11)				
Visit of other farm workers				
No (n = 1)	Ref			
Yes (n = 37)	-	-	1	1
Animal quarantine				
No (n = 35)	Ref			
Yes (n = 3)	-	-	1	1
Different age group animals kept at farm				
Separate (n = 37)	Ref			
Together (n = 1)	-	-	1	1
Farm hygiene				
Poor (n = 2)	Ref			
Good (n = 36)	-	-	1	1
Presence of other animal species other than bovine				
No (n = 37)	Ref			
Yes (n = 1)	-	-	1	1
Feeding method				
Stall feeding (n = 38)	Ref			
Grazing (n = 0)	-	-	1	1

OR=Odds ratio, CI=Confidence interval, df=Degree of freedom, p value=Fisher`s exact test, Ref=Reference

### Animal-level prevalence of FMD and associated risk factors:

**Figure-2:**
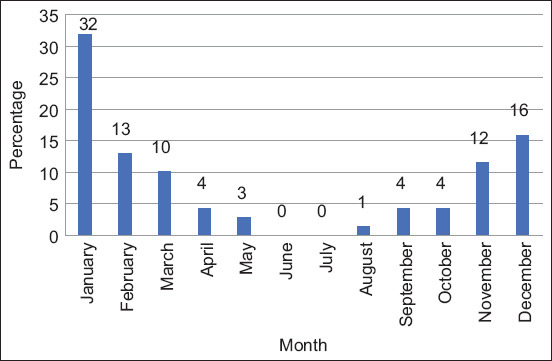
Cattle positive for foot-and-mouth disease from January to December 2021.

**Figure-3:**
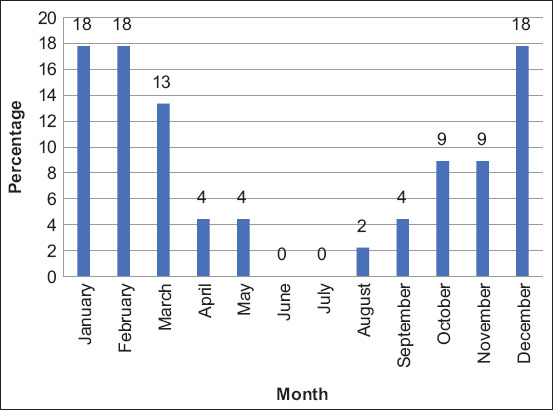
Buffalo positive for foot-and-mouth disease from January to December 2021.

**Figure-4:**
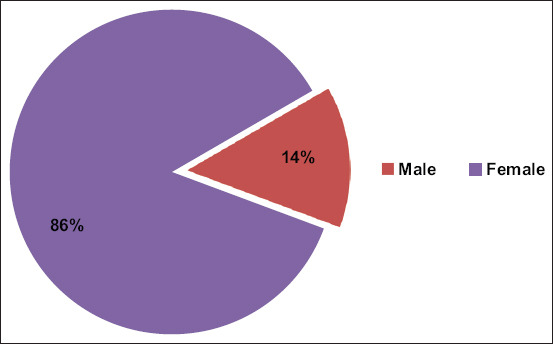
Male and female positive for foot-and-mouth disease.

**Figure-5:**
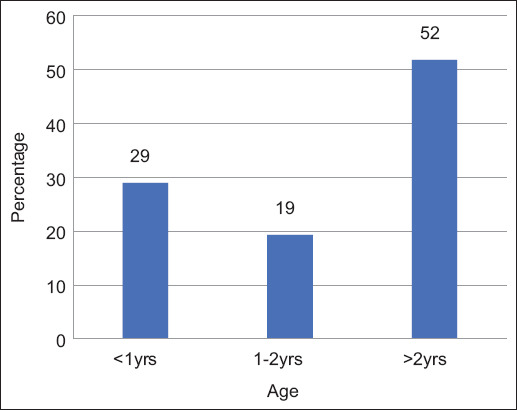
Animals positive for foot-and-mouth disease with respect to age groups.

Fifty-seven percent (114/200, 95% CI: 50%–64%) of animals were positive for FMD. Of these, 61% were cattle (69/114) and 39% were buffalo (45/114) (p = 0.2997). All FMD-positive animals were identified as serotype O. The highest number of positive cases was detected in January 2021, followed by a downward trend until May 2021. Further, an upward trend was observed from August 2021 till December 2021, both in cattle (Figure-2) and buffalo (Figure-3). FMD positivity was higher in females (86%, 98/114) than in males (14%, 16/114) (p = 0.438) (Figure-4). FMD infection was higher in animals with an age group of above 2 years (52%, 59/114), followed by <1 year (29%, 33/144) and 1–2 years (19%, 22/144) (p = 0.378) (Figure-5). All animal-related variables included in the univariate analysis showed an insignificant association with FMDV infection (Table-2).

**Table-2:** Univariable analysis of animal-related variables (n = 200).

Variable	OR	95% CI	df	p-value
Species				
Buffalo (n = 72)	Ref			
Cattle (n = 128)	0.71	0.37–1.32	1	0.297
Sex				
Female (n = 168)	Ref			
Male (n = 32)	0.71	0.31–1.64	1	0.438
Age				
< 1 year (n = 66)	Ref			
1-2 years (n = 37)	1.46	0.64–3.31	1	0.413
>2 years (n = 97)	1.55	0.82–2.92	1	0.199
Vaccination				
No (n = 35)	Ref			
Yes (n = 165)	1.14	0.51–2.53	1	0.851
Body condition				
Weak (n = 17)	Ref			
Healthy (n = 183)	0.92	0.28–2.82	1	1

OR=Odds ratio, CI=Confidence interval, df=Degree of freedom, p value=Fisher`s exact test, Ref=Reference

### Effect of FMD on hematological profile

White blood cell (WBC) concentrations were significantly higher in FMDV-infected cattle and buffaloes compared with non-infected animals (p < 0.05). Hb levels were significantly lower in FMDV-infected animals (p < 0.05). There were no differences in the concentrations of RBCs, MCV, HCT, MCHC, and platelets among FMDV-infected and non-infected cattle and buffaloes (Table-3).

**Table-3:** Effect of FMDV infection on hematological indices of cattle and buffalo.

Hematological indices	Cattle		Buffalo	
	
	Infected (n = 69)	Non-infected (n = 59)	Infected (n = 45)	Non-infected (n = 27)
RBCs (10^12^/L)	4.85 ± 0.18	5.18 ± 0.43	6.08 ± 0.29	6.09 ± 0.27
MCV (fL)	55.93 ± 2.83	51.16 ± 4.15	40.06 ± 1.10	38.85 ± 1.06
HCT (%)	20.24 ± 0.80	21.30 ± 1.73	22.36 ± 0.79	23.97 ± 0.78
Hb (mg/dL)	8.38 ± 0.32	9.42 ± 0.29*	9.16 ± 0.32	11.14 ± 0.55*
Platelets (10^9^/L)	229.43 ± 4.35	211.13 ± 6.15	222.37 ± 14.87	219.73 ± 16.72
WBCs (10^9^/L)	8.11 ± 0.44*	6.82 ± 0.52	8.02 ± 0.43*	6.56 ± 0.49
MCHC (mg/dL)	48.43 ± 2.33	45.52 ± 5.3	43.30 ± 2.29	42.06 ± 3.83

* p < 0.05, RBCs=Red blood cells, MCV=Mean corpuscular volume, HCT=Hematocrit test, Hb=Hemoglobin, WBCs=White blood cells, MCHC=Mean corpuscular hemoglobin concentration, fL=Femtoliters, %=Percentage, mg/dL=Milligrams per decilitre, FMDV=Foot-and-mouth disease virus

### Effects of FMD on biochemical parameters

Total protein concentrations were significantly lower in infected cattle and buffaloes (p < 0.05). Albumin levels were lower in the infected cattle (p < 0.05). Globulin levels were lower in buffaloes infected. ALT levels were lower in the infected cattle (p < 0.05). Creatinine levels were higher in the infected buffalo (p < 0.05). Urea and phosphorus concentrations were higher in FMDV-infected cattle and buffaloes (p < 0.05). Calcium levels were lower in infected cattle and buffaloes (p < 0.05). No differences in cholesterol, glucose, and AST levels were observed between FMDV-infected and non-infected cattle and buffalo (Table-4).

**Table-4:** Effect of FMDV infection on serum biochemistry of cattle and buffalo.

Serum biochemistry	Cattle		Buffalo	
	
	Infected (n = 69)	Non-infected (n = 59)	Infected (n = 45)	Non-infected (n = 27)
Total protein (g/dL)	8.29 ± 0.23	9.34 ± 0.48*	6.91 ± 0.3	8.52 ± 0.9*
Albumin (g/dL)	3.52 ± 0.07	4.35 ± 0.24*	3.25 ± 0.19	3.41 ± 0.12
Globulin (g/L)	4.74 ± 0.22	3.80 ± 0.34	3.66 ± 0.29	5.64 ± 0.95*
Cholesterol (mg/dL)	94.84 ± 2.65	100.43 ± 8.08	85.74 ± 4.23	94.55 ± 7.95
Glucose (mg/dL)	60.21 ± 2.1	57.47 ± 5.0	72.67 ± 4.67	61.92 ± 8.08
ALAT (U/L)	41.46 ± 1.60	64.06 ± 4.49*	30.90 ± 2.21	29.93 ± 3.89
ASAT (U/L)	62.35 ± 2.26	64.06 ± 4.49	56.70 ± 4.67	45.53 ± 7.92
Urea (mg/dL)	49.35 ± 1.15*	38.14 ± 2.51	53.97 ± 1.73*	43.18 ± 2.99
Creatinine (mg/dL)	1.615 ± 0.06	1.92 ± 0.17	2.60 ± 0.25*	1.55 ± 0.15
Calcium (mg/dL)	7.07 ± 0.17	8.55 ± 0.37*	6.34 ± 0.52	7.18 ± 0.50*
Phosphorus (mg/dL)	9.16 ± 0.18*	6.45 ± 0.64	9.51 ± 0.33*	7.65 ± 0.53

* p < 0.05, g/dL=Grams per deciliter, mg/dL=Milligrams per deciliter, ALT=Alanine aminotransferase, AST=Aspartate aminotransferase, U/L=Units per liter, ca=Calcium, P=Phosphorous, FMDV=Foot-and-mouth disease virus

### Effect of FMD on the oxidative status

Catalase enzyme activity in infected cattle and buffaloes was significantly lower (p < 0.05). Lipid peroxidation in FMDV-infected cattle and buffalo was significantly higher (p < 0.05) (Table-5).

**Table-5:** Effect of FMDV infection on oxidative status of cattle and buffalo.

Oxidative status	Cattle		Buffalo	
	
	Infected (n = 69)	Non-infected (n = 59)	Infected (n = 45)	Non-infected (n = 27)
Catalase enzyme activity (KU/L)	4.62 ± 0.42	7.76 ± 0.97*	3.33 ± 0.48	5.86 ± 0.91*
MDA (μmol/L)	1.78 ± 0.05*	1.25 ± 0.13	1.72 ± 0.04*	1.08 ± 0.18

* p < 0.05, MDA=Malondialdehyde, KU/L=Kilounits per litter, μmol/L=Micromole per litter, FMDV=Foot-and-mouth disease virus

## Discussion

FMD is endemic in Pakistan with seasonal outbreaks. It is considered to be one of the major transboundary diseases affecting livestock in the country [17]. The Quetta district is geographically located in the north-west part of Balochistan province, close to the Afghanistan border, with livestock movement across the border and, consequently, a high probability of transboundary animal diseases [18]. FMD is a highly contagious transboundary viral disease that causes economic loss to livestock farmers and subsequently increases food insecurity [19]. The Quetta district has the highest number of human populations in the province, which is dependent on livestock as a protein source. Therefore, this study was designed and conducted to determine the detection of FMDV serotypes in year-round FMD outbreaks, hematological and biochemical changes, and oxidative stress in FMDV-infected cattle and buffaloes in the district of Quetta, Balochistan, Pakistan, and the socioeconomic impact of FMD outbreaks on farmers.

In the present study, a higher number of FMD infections were reported in farms with animal populations of <50, no quarantine practice for newly purchased animals, and visitors from other farms. A previous study by Ali *et al*. [17] in the Punjab province of Pakistan reported a large herd size as an associated risk factor for FMD outbreak, in contradiction to our findings. In the same study, the introduction of new animals in the herd with unknown vaccination status and the visit of animal brokers to the farm were associated risk factors for FMD outbreak, consistent with the findings of our study. The implementation of biosecurity on farms is essential to prevent the introduction of any disease to the farm. As a result, any lack of biosecurity can cause FMD outbreaks on farms and, consequently, significant economic losses for livestock farmers.

In the present study, FMD outbreaks occurred in livestock farms irrespective of regular FMD vaccination. Similar findings have been reported in a previous study by Singh *et al*. [20] in India, where FMD outbreaks were reported despite mass vaccination. To ensure effective FMD vaccination, it is necessary to follow an appropriate vaccination schedule, maintain cold storage, and most importantly, vaccinate the animals against the prevalent circulating FMD strain in the area. Non-compliance leads to FMD outbreaks in any area despite vaccination, which could be a possible reason for these outbreaks in the present study area.

The highest number of FMD-positive cases was detected in January 2021, followed by a downward trend until May 2021. Furthermore, an upward trend was observed between August 2021 and December 2021 in cattle and buffalo. Therefore, in the present study, the highest number of FMD cases was reported in the winter season of the study area. Similar findings have been reported in previous studies by Ali *et al*. [17], and Klein *et al*. [21] from Pakistan and other parts of the world. Seasonal FMD outbreaks in the study area could be used as an early warning tool to prevent the disease by adopting all possible preventive practices in advance to prevent FMD outbreaks and, consequently, economic loss to farmers.

Fifty-seven percent of animals were positive for FMD in the current study. Similar findings have been reported in a previous study by Klein *et al*. [21] among livestock. Another study conducted in Pakistan reported 33%, which is much lower than our findings [22]. The prevalence of FMD differs from country to country and is mainly dependent on the geographical environment, climatic conditions, and preventive measures implemented in the relevant area.

In the current study, FMD positivity was higher in cattle compared to buffalo. Similar findings have also been reported in previous studies by Jamal *et al*. [19], Abubakar *et al*. [23], and Abubakar *et al*. [24] in Pakistan. Cattle and buffaloes are cloven-hoofed animals that are equally susceptible to FMDV infection. This difference may be due to the higher exposure of cattle to FMDV infection compared with buffalo.

In the current study, all FMD-positive animals were detected as serotype O. Previous studies by Abubakar *et al*. [22], and Stenfeldt *et al*. [25] in Pakistan have reported serotype O as the most prevalent FMDV serotype in the country. To prevent the outbreak of FMD in livestock, the vaccine used in the study area should be tested for its efficacy against this serotype.

The current study showed no significant difference in FMDV infection between the various age groups. Similar findings have been reported in previous studies by Gelaye *et al*. [26], and Belina *et al*. [27]. However, some studies have reported significant differences between different age groups of animals with FMDV infection [28]. All age groups are susceptible to FMDV infection. As mentioned above, higher infection in any age group may be associated with higher exposure of that age group to FMDV infection.

Most of the hematological parameters were unaffected by FMDV infection, except for increased and decreased levels of WBCs and Hb in FMDV-infected animals, respectively. Similar findings have also been reported in previous studies by Gökçe *et al*. [29], El-Amir *et al*. [30], and El-Deen *et al*. [31]. The WBC level usually increases in almost every infection. The decrease in Hb levels affects the general health of animals and consequently reduces production, particularly in dairy animals.

In the current study, the total protein level was significantly lower in FMDV-infected animals. This is consistent with the findings of a previous study by Faruk *et al*. [32]. In addition, higher levels of urea and phosphorus and lower levels of calcium have been detected in FMDV-infected animals. These results are in agreement with previous studies by El-Deen *et al*. [31] and Saravanan *et al*. [33]. Variations in these blood parameters indicate that FMDV affects the major organs of animals, as previously reported by Meyer and Harvey [34] and hepatic and renal damage decreases total protein levels.

In the present study, a lower level of catalase enzyme and a higher level of lipid peroxidation were detected in FMDV-infected animals. Similar findings have also been reported in previous studies by El-Deen *et al*. [31] and Soltani *et al*. [35]. Both of them have important functions in the animal body.

## Conclusion

This study confirms the circulation of serotype O among cattle and buffalo in year-long FMD outbreaks in Quetta district of Balochistan province. The highest number of FMD cases was detected in the winter season and in farms with <50 animals. Animals of all age groups were susceptible to FMDV infection. Blood analysis showed that the parameter deviated from the normal level due to FMDV infection. In addition, FMD outbreaks have a significant negative economic impact on livestock farmers.

## Authors’ Contributions

DK, ISS, AU, and KKK: Conceived and designed the study. DK, MZM, ZUD, IA, and NK: Collected the samples and analyzed the data. DK, KKK, and NK: Performed the experiments. DK, KKK, and AW: Analyzed and interpreted the data and drafted and revised the manuscript. All authors have read, reviewed, and approved the final manuscript.
